# An Efficient Method for the Preparative Isolation and Purification of Flavonoid Glycosides and Caffeoylquinic Acid Derivatives from Leaves of *Lonicera japonica* Thunb. Using High Speed Counter-Current Chromatography (HSCCC) and Prep-HPLC Guided by DPPH-HPLC Experiments

**DOI:** 10.3390/molecules22020229

**Published:** 2017-02-02

**Authors:** Daijie Wang, Ning Du, Lei Wen, Heng Zhu, Feng Liu, Xiao Wang, Jinhua Du, Shengbo Li

**Affiliations:** 1 Shandong Analysis and Test Center, Shandong Academy of Sciences, 19 Keyuan Street, Jinan 250014, China; wangdaijie@126.com (D.W.); 18663081657@163.com (L.W.); sdzbzdzh@sina.com (H.Z.); liufeng8109@163.com (F.L.); 2 Beijing Centre for Physical and Chemical Analysis, Beijing 100089, China; maggieduning@163.com; 3 College of Food Science and Engineering, Shandong Agricultural University, 61 Daizong Street, Taian 271018, China; 4 Shandong Yate Eco-Tech Co. Ltd., Linyi 266071, China; yate886@sohu.com

**Keywords:** *Lonicera japonica* Thunb., DPPH-HPLC experiments, flavonoid glycosides, caffeoylquinic acid derivatives, high speed counter-current chromatography, prep-HPLC combination

## Abstract

In this work, the *n*-butanol extract from leaves of *Lonicera japonica* Thunb. (*L. japonica*) was reacted with DPPH and subjected to a HPLC analysis for the guided screening antioxidants (DPPH-HPLC experiments). Then, nine antioxidants, including flavonoid glycosides and caffeoylquinic acid derivatives, were isolated and purified from leaves of *L. japonica* using high speed counter-current chromatography (HSCCC) and prep-HPLC. The *n*-butanol extract was firstly isolated by HSCCC using methyl *tert*-butyl ether/*n*-butanol/acetonitrile/water (0.5% acetic acid) (2:2:1:5, *v/v*), yielding five fractions F1, F2 (rhoifolin), F3 (luteoloside), F4 and F5 (collected from the column after the separation). The sub-fractions F1, F4 and F5 were successfully separated by prep-HPLC. Finally, nine compounds, including chlorogenic acid (**1**), lonicerin (**2**), rutin (**3**), rhoifolin (**4**), luteoloside (**5**), 3,4-*O*-dicaffeoylquinic acid (**6**), hyperoside (**7**), 3,5-*O*-dicaffeoylquinic acid (**8**), and 4,5-*O*-dicaffeoylquinic acid (**9**) were obtained, respectively, with the purities over 94% as determined by HPLC. The structures were identified by electrospray ionization mass spectrometry (ESI-MS), ^1^H- and ^13^C-NMR. Antioxidant activities were tested, and the isolated compounds showed strong antioxidant activities.

## 1. Introduction

*Lonicera japonica* Thunb., one of the most common traditional Chinese medicines, is widely used for treating various diseases, including arthritis, diabetes mellitus, fever, infections, sores and swelling. Pharmacological studies have demonstrated that their extracts have a broad spectrum of biological activities, such as antibacterial, anti-inflammatory, antioxidant, antipyretic, antiviral, antimicrobial, and hepatoprotective effects [1,2,3,4].

Leaves of *L. japonica* are the offal of this plant. The production is five times larger than that of flowers. Many studies have indicated that their extracts have antioxidant and bacteriostatic activity and might be a potential source of preservatives for use in the food or pharmaceutical industries [5,6,7,8], so it is urgent to establish an efficient method for the preparative isolation and purification of compounds from leaves of *L. japonica*.

A number of conventional separation methods (e.g., silica gel, polyamide column chromatography, macroporous resin and Sephadex LH-20) are used for separating compounds from *L. japonica*. Because of the similarities between the chemical structures and their instability, these methods have many disadvantages, such as irreversible sample adsorption, the need for complex multiple steps and large solvent consumption.

High-speed counter-current chromatography (HSCCC) is a support free liquid-liquid partition chromatography which can avoid the irreversible adsorption of the sample onto a solid support. Due to the advantages of its separation principle such as high recovery rate, simple sample preparation, large sample injection and excellent repeatability, HSCCC has been used to separate and isolate various samples [9,10,11,12,13,14,15]. Preparative high-performance liquid chromatography (prep-HPLC) is a powerful tool in virtue of its excellent separation efficiency. However, it is more expensive and typically requires sample treatment before separation, as raw samples will quickly contaminate and overload the columns. Considering a possible complementarity between HSCCC and prep-HPLC, in the present paper, a method combining HSCCC and prep-HPLC was established for the rapid separation and purification of compounds from leaves of *L. japonica*. In previous studies, caffeoylquinic acid derivatives from *L. japonica* bus were separated with HSCCC and prep-HPLC [16,17], yet complex flavonoid glycosides were not reported in the materials.

**Figure 1:**
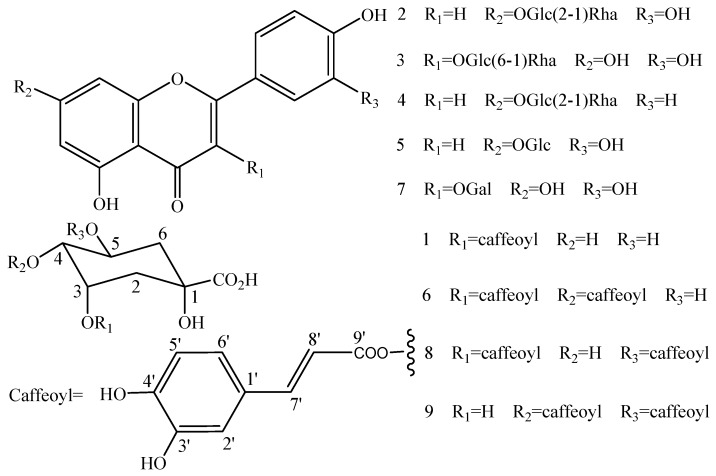
Chemical structures of the nine compounds.

The present study is the first to use HSCCC and prep-HPLC guided by DPPH-HPLC experiments for the screening and preparative separation of flavonoid glycosides and caffeoylquinic acid derivatives from the leaves of *L. japonica*. Nine compounds were separated and their chemical structures were elucidated by electrospray ionization mass spectrometry (ESI-MS) and NMR. Their chemical structures are shown in Figure 1.

## 2. Results and Discussion

### 2.1. Screen Antioxidants by DPPH-HPLC Analysis 

The DPPH-HPLC method has been used for rapid screening of the radical scavenging properties of natural products [18]. A reduced or absent peak in the HPLC chromatogram after reaction with DPPH indicates potential antioxidant compounds. Peaks with no changes are considered without antioxidant abilities.

**Figure 2:**
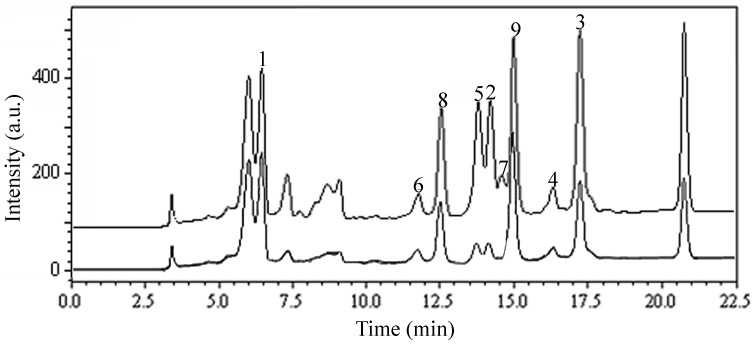
DPPH-HPLC chromatograms of *n*-butanol extract of *Lonicera japonica* leaves. Experimental conditions: Shim-pack VP-ODS column (250 mm × 4.6 mm, i.d., 5 μm); Column temperature: 25 °C; Flow rate: 1.0 mL/min; Detection: 254 nm; Injection volume: 10 μL. HPLC conditions are as follows: eluent A (MeOH) and eluent B (0.3% acetic acid in water, *v*/*v*), linear gradient combinations: at *t* = 0, 70% B; at *t* = 20 min, 40% B; at *t* = 22.5 min, 40% B. a.u.: arbitrary unit.

The *n*-butanol extract from leaves of *L. japonica* showed potential capacity to scavenge DPPH radical with an IC_50_ value of 11.2 µM (Table 1). This indicated that there were abundant antioxidant compounds in the *n*-butanol extract. Then, the DPPH-HPLC method was used to scan them. The chromatogram, detected at 254 nm, of the *n*-butanol extract that reacted with DPPH is shown in Figure 2. Seven of the compounds **1**–**9** in the extract were prominently reduced, indicating they possessed antioxidant activity. Based on the ratio of the reduced peak area, compounds **2**, **4**, **5** and **7** were the major compounds with antioxidant activity.

### 2.2. Optimization of HSCCC Conditions

The major challenge of HSCCC separation is the selection of the correct solvent system, which can account for 90% of the time spent on the analysis [19,20]. Successful separation by HSCCC requires a suitable *K*_D_-value (partition coefficient). Large *K*_D_-values usually tend to produce excessive sample band broadening, while small *K*_D_-values result in poor peak resolution [21]. As flavonoid glycosides and caffeoylquinic acid derivatives are highly polar compounds, several hydrophilic two-phase solvent systems were tested in this study and their *K*_D_-values were measured and summarized in Table 2.

**Figure 3:**
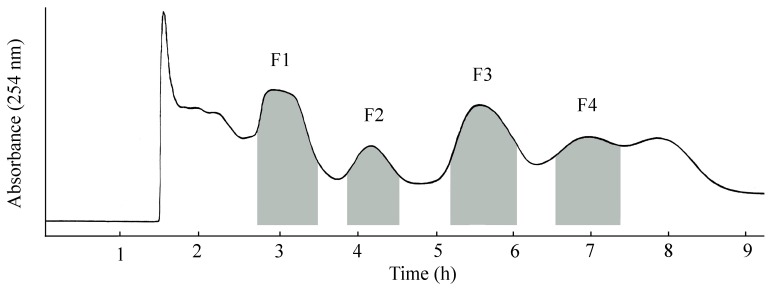
Chromatogram of the crude extract by HSCCC. Two-phase solvent system: methyl *tert*-butyl ether/*n*-butanol/acetonitrile/water (0.5% acetic acid) (2:2:1:5, *v/v*); Mobile phase: the lower phase; Flow rate: 2.0 mL/min; Revolution speed: 850 rpm; Detection wavelength: 254 nm; Sample size: 1.0 g; Injection volume: 10 mL; Retention of stationary phase: 47.3%; F2: rhoifolin; F3: luteoloside.

When *n*-hexane/*n*-butanol/methanol/water (1:3:1:4, *v/v*) and (1:4:1:4, *v/v*) were used as the two-phase solvent system, suitable *K*_D_-values could be obtained. When these two-phase solvent systems were used for separation, the retention of the stationary phase was poor (<25%) and the amount of sample was not large enough. When methyl *tert*-butyl ether/*n*-butanol/acetonitrile/water (0.5% acetic acid) (1.5:2.5:1:5, *v/v*) was used as the two-phase solvent system, the *K*_D_-value was too big and would result in a long separation time. Among these solvents, methyl *tert*-butyl ether/*n*-butanol/acetonitrile/water (0.5% acetic acid) (2.5:1.5:1:5, *v/v*) and (2:2:1:5, *v/v*) were suitable for the separation. When methyl *tert*-butyl ether/*n*-butanol/acetonitrile/water (0.5% acetic acid) (2.5:1.5:1:5, *v/v*) was used as the two-phase solvent system, the close HSCCC peaks were not fully resolved. After trying methyl *tert*-butyl ether/*n*-butanol/acetonitrile/water (0.5% acetic acid) (2:2:1:5, *v/v*), it was found to be the best to effect the separation. Figure 3 shows the chromatogram separated by HSCCC using this solvent system.

The *n*-butanol extract (1.0 g) from leaves of *L. japonica* was purified under the optimum HSCCC conditions. The upper phase was used as the stationary phase while the lower was the mobile phase in the head to tail elution mode. The retention of the stationary phase was 47.3%, and the total separation time was about 9 h. Then, the HSCCC fractions were analyzed by HPLC, and their absorbance was measured at 254 nm to draw the elution curve (Figure 3). Based on the HPLC analysis, five sub-fractions F1 (69.7 mg), F2 (21.5 mg), F3 (31.9 mg), F4 (148.7 mg) and F5 (62.8 mg, collected from the column after the separation) were obtained. F1 was a mixture of chlorogenic acid, lonicerin and rutin. F2 and F3 were rhoifolin and luteoloside, with purities of 94.3% and 96.1%, respectively, as determined by HPLC. F4 was a mixture of 3,4-*O*-dicaffeoylquinic acid and hyperoside. F5 (mixed with 3,5-*O*-dicaffeoylquinic acid and 4,5-*O*-dicaffeoylquinic acid) was fraction collected by blowing out the column solution after separation because the *K*_D_-values of compounds **8** and **9** were too big and would result in a long time separation. The sub-fractions F1, F4 and F5 were further subjected to preparative HPLC separation.

### 2.3. Prep-HPLC Separation 

**Figure 4:**
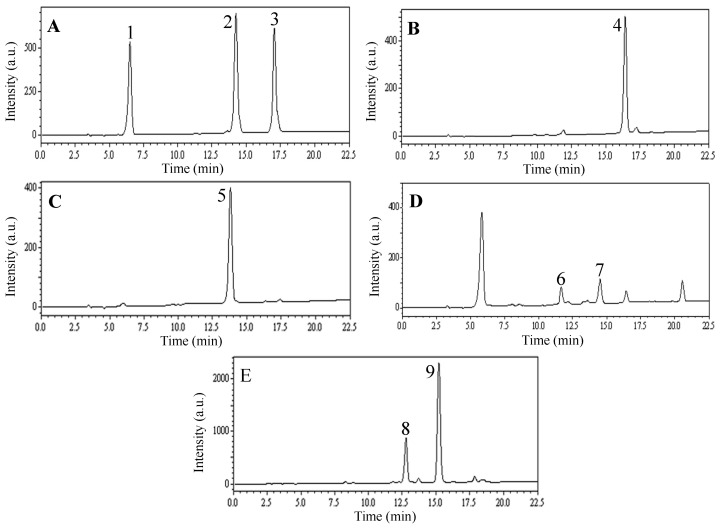
HPLC chromatograms of *n*-butanol extract and HSCCC peak fractions from leaves of *L. japonica*. ((**A**) F1 in Figure 3; (**B**) F2 in Figure 3; (**C**) F3 in Figure 3; (**D**) F4 in Figure 3; (**E**) F5 collected from the column after the separation). Experimental conditions: Shim-pack VP-ODS column (250 mm × 4.6 mm, i.d., 5 μm); Column temperature: 25 °C; Flow rate: 1.0 mL/min; Detection: 254 nm; Injection volume: 10 μL. HPLC conditions are as follows: Eluent A (MeOH) and eluent B (0.3% acetic acid in water, *v/v*), linear gradient combinations: at *t* = 0, 70% B; at *t* = 20 min, 40% B; at *t* = 22.5 min, 40% B. a.u.: arbitrary unit.

**Figure 5:**
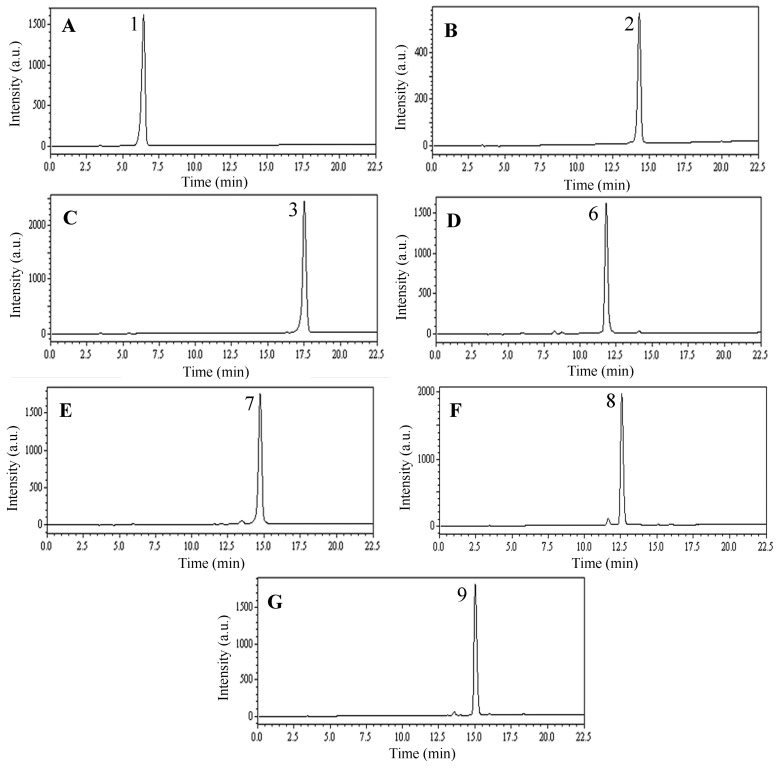
HPLC chromatograms of prep-HPLC peak fractions. ((**A**) chlorogenic acid, compound **1** in Figure 4A; (**B**) lonicerin, compound **2** in Figure 4A; (**C**) rutin, compound **3** in Figure 4A; (**D**) 3,4-*O*-dicaffeoylquinic acid, compound **6** in Figure 4D; (**E**) hyperoside, compound **7** in Figure 4D; (**F**) 3,5-*O*-dicaffeoylquinic acid, compound **8** in Figure 4E; (**G**) 4,5-*O*-dicaffeoylquinic acid, compound **9** in Figure 4E). a.u.: arbitrary unit.

The HPLC analyses of F1, F4 and F5 are shown in Figure 4. The preparative HPLC separations of F1, F4 and F5 finally yielded seven compounds corresponding to peak 1 of Figure 4A (10.9 mg of chlorogenic acid), peak 2 of Figure 4A (30.7 mg of lonicerin), peak 3 of Figure 4A (16.7 mg of rutin), peak 6 of Figure 4A (20.3 mg of 3,4-*O*-dicaffeoylquinic acid), peak 7 of Figure 4A (18.2 mg of hyperoside), peak 8 of Figure 4A (24.7 mg of 3,5-*O*-dicaffeoylquinic acid) and peak 9 of Figure 4A (26.1 mg of 4,5-*O*-dicaffeoylquinic acid), with purities of 99.5%, 98.7%, 99.3%, 97.1%, 97.4%, 96.9% and 97.8%, respectively, as determined by HPLC. Table 3 lists the preparative HPLC separation conditions of F1, F4 and F5 and Figure 5 shows the chromatograms of the individual components.

### 2.4. Antioxidant Activities of Isolated Compounds

The antioxidant activity of flavonoid glycosides and caffeoylquinic acid derivatives depend on the number and position of hydroxyl groups bound to the aromatic ring, the binding sites and the mutual positions of hydroxyl groups in the aromatic ring and the type of substituent [22]. In the DPPH radical scavenging assays of the compounds isolated from the leaves of *L.* *japonica*, ascorbic acid and vitamin E were selected as the standard antioxidants. As shown in Table 1, compounds **1**–**9** exhibited strong DPPH radical activities with IC_50_ values less than 12 µg/mL. The presence of these flavonoid glycosides suggest a possible commercial value and recommend the further development of *L*. *japonica* leaves as a source of anti-oxidant compounds.

## 3. Materials and Method

### 3.1. Apparatus and Materials

HSCCC was carried out using a Model TBE-300A commercial instrument (Tauto Biotech, Shanghai, China). The apparatus was equipped with three PTFE preparative coils (internal diameter of tube: 1.6 mm, total volume: 280 mL) and a 20 mL sample loop. The *β* values of this preparative column range from 0.47 at the internal to 0.73 at the external (*β = r*/*R*, where *r* is the rotation radius or the distance from the coil to the holder shaft, and *R* (*R* = 7.5 cm) is the revolution radius or the distances between the holder axis and central axis of the centrifuge). The solvent was pumped into the column with a Model NS-1007 constant-flow pump (Beijing Emilion Science & Technology Co., Beijing, China). Continuous monitoring of the effluent was carried out with a Model 8823A-UV detector (Beijing Emilion Science & Technology Co.). A Model 3057 portable recorder (Yokogawa, Sichuan Instrument Factory, Chongqing, China) was used to plot the chromatogram.

The HPLC analysis used throughout this study consisted of a Waters 996 photodiode array detection, a Waters 600 Multisolvent Delivery, a Waters 600 system controller, a Waters 600 pump, and a Millennium 32 workstation (Waters, Milford, CT, USA). The prep-HPLC used throughout this study consisted of Shimadzu SPD M20A photodiode array detection, a Shimadzu CBM-20A system controller, a Shimadzu LC-6AD pump, and an LC-solution workstation (Shimadzu, Kyoto, Japan).

*n*-Hexane, methyl *tert*-butyl ether, *n*-butanol, acetonitrile, ethyl acetate, methanol, acetic acid and ethanol used for extraction and separation were of analytical grade (Sinopharm Chemical Reagent Co., Ltd, Shanghai, China). Methanol used for HPLC analysis was of chromatographic grade (Merck, Darmstadt, Germany). Reverse osmosis Milli-Q water (Millipore, Billerica, MA, USA) was used for all solutions and dilutions.

### 3.2. Plant Material

The leaves of *L. japonica* were obtained from Shandong Yate Eco-tech Co., LTD and identified by Dr. Li Jia (College of Pharmacy, Shandong University of Traditional Chinese Medicine, Jinan, China).

### 3.3. Preparation of Extracts

The dried leaves of *L. japonica* were ground to a powder. The powder (1 kg) was extracted three times with 80% ethanol by the heating circumfluence method (5 L and 2 h per time).The extracts were combined and concentrated under reduced pressure at 60 °C until the ethanol had been removed. The residue was diluted with water (2 L) and extracted five times with ethyl acetate and *n*-butanol in room temperature (2 L and 10 min per time). A total of 35.5 g of *n*-butanol extract was obtained for the further separation and purification.

### 3.4. Evaluation of Antioxidant Activity

The DDPH radical assay was performed based on Tapia’s method [23]. Diluted sample (25 µL) was mixed with DPPH solution (40 µL, 0.4 mg/mL) which was served as a control, then, filled up with methanol to 250 µL. The mixtures were incubated at 37 °C for 30 min, and then measured at 517 nm. The antioxidant activities were expressed as the percentage of DPPH radical elimination which was calculated from the formula: [(*A*_blank_ − *A*_sample_)/*A*_blank_] × 100%, where *A*_blank_ and *A*_sample_ were the absorbance of black DPPH solution with addition of sample, respectively. Sample concentration providing 50% inhibition (IC_50_) was calculated from the graph plotting the inhibition percentage. Every test was run for three times, meanwhile the average value was calculated.

### 3.5. DPPH-HPLC Experiment

The *n*-butanol extract from leaves of *L. japonica* was reacted with DPPH by the same procedure as described in Section 3.4. The mixtures were passed through a 0.45 µm filter for HPLC analysis. A blank of aqueous extraction was used as a control. Analytical HPLC was carried out with a Waters 996 photodiode array detection (PDA), a Waters 600 Multisolvent Delivery, a Waters 600 system controller, and a Waters 600 pump. The separation was performed on a Shim-pack VP-ODS column (250 mm × 4.6 mm, i.d., 5 μm) (Shimadzu, Kyoto, Japan); gradient elution was performed using an A eluent (MeOH) and a B eluent (0.3% acetic acid in water, *v*/*v*) with the following linear gradient combinations: at t = 0, 70% B; at t = 20 min, 40% B; at t = 22.5 min, 40% B. Flow rate was 1.0 mL/min, and 10 μL portion was injected into the column. The effluent was monitored at 254 nm.

### 3.6. Selection of Two-Phase Solvent System

The two-phase solvent system was selected according to the partition coefficient (*K*_D_) of the target compound of the samples. The partition coefficient was determined by HPLC as follows: 10 mg of the crude extract was dissolved in the 2 mL lower phase of the two-phase solvent system. The solution was then determined by HPLC. The peak area was recorded as *A*_1_. Then equal volume of the upper phase was added to the solution and mixed thoroughly. After the partition equilibration was reached, the lower phase solution was determined by HPLC again, and the peak area was recorded as *A*_2_. The *K*_D_-value was defined as the following equation: *K*_D_ = (*A*_1_ − *A*_2_)/*A*_2_ [24].

### 3.7. Preparation of the Two-Phase Solvent System and Sample Solution

The selected two-phase solvent system methyl *tert*-butyl ether/*n*-butanol/acetonitrile/water (0.5% acetic acid) (2:2:1:5, *v/v*) was used in the HSCCC experiments. After thoroughly equilibrating the solvents in a separation funnel, the two phases were separated shortly before use. The lower phase was used as mobile phase, while the upper organic was used as the stationary phase. The sample solution was prepared by dissolving the crude sample in 10 mL of stationary phase and mobile phase (1:1).

### 3.8. HSCCC Separation Procedure

Preparative HSCCC was performed as follows: the multilayer coiled column was first entirely filled with the upper phase, and then the lower phase was pumped into the head end of the column inlet at a flow rate of 2.0 mL/min, while the column was rotated at 850 rpm. After the mobile phase front emerged and hydrodynamic equilibrium was established, the sample solution (1.0 g dissolved in 10 mL mixture solvent system) was injected through the sample port. The effluent from the outlet of the column was continuously monitored by UV detector at 254 nm, and the peak fractions were collected according to the recorded elution profile. The retention of the stationary phase relative to the total column capacity was computed from the volume of the stationary phase collected from the column after the separation was completed.

### 3.9. HPLC Analyses of HSCCC and prep-HPLC Peak Fractions

HPLC conditions were as follows: Shim-pack VP-ODS column (250 mm × 4.6 mm, i.d., 5 μm); gradient elution performed using an A eluent (MeOH) and a B eluent (0.3% acetic acid in water, *v/v*) with the following linear gradient combinations: at t = 0, 70% B; at t = 20 min, 40% B; at t = 22.5 min, 40% B. Flow rate was 1.0 mL/min, and 10 μL portion was injected into the column. The effluent was monitored at 254 nm. Prep-HPLC separations were performed on a Shim-pack PREP-ODS column (20.0 mm × 250 mm, 15 μm) (Shimadzu, Kyoto, Japan); The mobile phase, a solution of eluent A (MeOH) and eluent B (0.3%, *v/v*, acetic acid in water), was monitored at 254 nm. The processing conditions for the linear gradient elution of F1, F4 and F5 are listed in Table 3.

### 3.10. Electrospray Ionization Mass Spectrometry and NMR

The identification of HSCCC peak fractions was performed by mass spectrometry (ESI-MS) with an Agilent 1100/MSD (Agilent, Santa Clara, CA, USA), and NMR spectra with a Varian-600 spectrometer (Varian, Palo Alto, CA, USA) with tetramethylsilane (TMS) as internal standard.

### 3.11. Identification of the Isolated Compounds

The structural identification of compound was performed with ESI-MS (operated both in the negative and positive ion mode in the range of *m/z* 100–1000), ^1^H- and ^13^C-NMR spectra with TMS as internal standard.

*Chlorogenic acid* (**1**, Figure 5A): ESI-MS, *m/z* 353.3 [M − H]^−^. ^1^H-NMR (600 MHz, DMSO-*d*_6_) δ: 7.42 (1H, d, *J* = 15.0 Hz, H-7′), 7.05 (1H, br s, H-2′), 6.99 (1H, br d, *J* = 7.8 Hz, H-6′), 6.77 (1H, d, *J* = 8.4 Hz, H-5′), 6.15 (1H, d, *J* = 15.0 Hz, H-8′), 5.07 (1H, m, H-3), 3.93 (1H, m, H-5), 3.42 (1H, m, H-4), 1.77–2.02 (4H, m, H-2, 6). ^13^C-NMR (125 MHz, DMSO-*d*_6_) δ: 175.0 (C-7), 165.8 (C-9′), 148.4 (C-4′), 145.6 (C-3′), 145.0 (C-7′), 125.6 (C-1′), 121.4 (C-6′), 115.8 (C-5′), 114.8 (C-2′), 114.3 (C-8′), 73.5 (C-1), 70.9 (C-5), 70.3 (C-4), 68.0 (C-3), 37.2 (C-2), 36.2 (C-6). Comparing the data with reference [25], the obtained compound was identified as chlorogenic acid.

*Lonicerin* (**2**, Figure 5B): ESI-MS, *m/z* 595.0 [M + H]^+^, 593.1 [M − H]^−^. ^1^H-NMR (600 MHz, DMSO-*d*_6_) δ: 9.45, 9.41, 13.02 (3H, s, OH × 3), 7.44 (1H, d, *J* = 7.2 Hz, H-2′), 7.42 (1H, dd, *J* = 1.8, 7.2 Hz, H-6′), 6.90 (1H, d, *J* = 7.8 Hz, H-5′), 6.77 (1H, s, H-3), 6.75 (1H, d, *J* = 1.2 Hz, H-8), 6.38 (1H, d, *J* = 1.8 Hz, H-6), 5.26 (1H, d, *J* = 7.8 Hz, H- H-1″), 5.14 (1H, s, H-1′″), 1.21 (3H, d, *J* = 6.6 Hz, 6′″-CH_3_). ^13^C-NMR (125 MHz, DMSO-*d*_6_) δ: 181.7 (C-4), 164.4 (C-2), 162.4 (C-7), 161.0 (C-5), 156.9 (C-9), 150.0 (C-4′), 145.7 (C-3′), 121.1 (C-1′), 119.0 (C-6′), 115.9 (C-5′), 113.4 (C-2′), 105.3 (C-10), 103.0 (C-3), 100.3 (rha C-1), 99.1 (glc C-1), 97.6 (C-6), 94.2 (C-8), 77.0 (glc C-2), 76.9 (glc C-3), 76.1 (glc C-5), 71.7 (rha C-4), 70.4 (rha C-3), 70.3 (rha C-2), 69.5 (glc C-4), 68.2 (rha C-5), 60.3 (glc C-6), 18.0 (rha C-6). Comparing the data with reference [26], the obtained compound was identified as lonicerin.

*Rutin* (**3**, Figure 5C): ESI-MS, *m/z* 611.1 [M + H]^+^, 609.0 [M − H]^−^. ^1^H-NMR (600 MHz, DMSO-*d*_6_) δ: 9.18, 9.66, 10.81, 12.59 (4H, s, OH × 4), 6.18 (1H, d, *J* = 1.8 Hz, H-6), 6.36 (1H, d, *J* = 2.4 Hz, H-8), 6.83 (1H, d, *J* = 8.4 Hz, H-5′), 7.54 (1H, dd, *J* = 1.8, 8.4 Hz, H-6′), 7.51 (1H, d, *J* = 2.4 Hz, H-2′). ^13^C-NMR (125 MHz, DMSO-*d*_6_) δ: 177.3 (C-4), 164.0 (C-7), 161.2 (C-5), 156.6 (C-9), 156.5 (C-2), 148.5 (C-4′), 144.7 (C-3′), 133.3 (C-3), 121.6 (C-1′), 121.1 (C-6′), 116.2 (C-5′), 115.1 (C-2′), 103.9 (C-10), 101.2 (glc C-1), 100.7 (rha C-1), 98.5 (C-6), 93.5 (C-8), 76.5 (glc C-3), 76.1 (glc C-5), 74.2 (glc C-2), 71.9 (rha C-4), 70.6 (glc C-4), 70.4 (rha C-3), 70.2 (rha C-2), 68.2 (rha C-5), 67.0 (glc C-6), 17.8 (rha C-6). Comparing the data with reference [27], the obtained compound was identified as rutin.

*Rhoifolin* (**4**, Figure 4B): ESI-MS, *m/z* 579.4 [M + H]^+^, 577.4 [M − H]^−^. ^1^H-NMR (600 MHz, CD_3_OD) δ: 7.85 (2H, d, *J* = 8.4 Hz, H-2′, 6′), 6.91 (2H, d, *J* = 8.4 Hz, H-3′, 5′), 6.74 (1H, br s, H-8), 6.63 (1H, s, H-3), 6.43 (1H, br s, H-6), 5.29 (1H, s, H-1′″), 5.18 (1H, d, *J* = 6.6 Hz, H-1″), 1.33 (3H, d, *J* = 6.6 Hz, 6′″-CH_3_). ^13^C-NMR (125 MHz, CD_3_OD) δ: 184.0 (C-4), 166.7 (C-2), 164.4 (C-7), 163.0 (C-5), 162.9 (C-4′), 158.9 (C-9), 129.7 (C-2′, 6′), 123.0 (C-1′), 117.1 (C-3′, 5′), 107.1 (C-10), 104.1 (C-3), 102.6 (rha C-1), 101.0 (C-6), 99.8 (glc C-1), 95.9 (C-8), 79.0 (glc C-2, 3), 78.3 (glc C-5), 74.0 (rha C-4), 72.2 (rha C-2, glc C-4), 71.4 (rha C-3), 70.0 (rha C-5), 62.4 (glc C-6), 18.2 (rha C-6). Comparing the data with reference [25], the obtained compound was identified as rhoifolin.

*Luteoloside* (**5**, Figure 4C): ESI-MS, *m/z* 449.3 [M + H]^+^. ^1^H-NMR (600 MHz, DMSO-*d*_6_) δ: 9.43, 10.03, 13.00 (3H, s, OH × 3), 7.43–7.45 (2H, m, H-2′, 6′), 6.92 (1H, d, *J* = 8.4 Hz, H-5′), 6.80 (1H, d, *J* = 2.4 Hz, H-8), 6.77 (1H, s, H-3), 6.45 (1H, d, *J* = 2.4 Hz, H-6), 5.10 (1H, d, *J* = 7.2 Hz, H-1″). ^13^C-NMR (125 MHz, DMSO-*d*_6_) δ: 181.9 (C-4), 164.4 (C-2), 162.9 (C-7), 161.1 (C-5), 156.9 (C-9), 149.9 (C-4′), 145.8 (C-3′), 121.4 (C-1′), 119.2 (C-6′), 116.0 (C-5′), 113.6 (C-2′), 105.3 (C-10), 103.2 (C-3), 99.8 (C-6), 99.8 (glc C-1), 94.7 (C-8), 77.1 (glc C-5), 76.4 (glc C-3), 73.1 (glc C-2), 69.5 (glc C-4), 60.6 (glc C-6). Comparing the data with reference [28], the obtained compound was identified as luteoloside. 

*3,4-O-Dicaffeoylquinic acid* (**6**, Figure 5D): ESI-MS, *m/z* 539.0 [M + Na]^+^, 515.1 [M − H]^−^. ^1^H-NMR (600 MHz, CD_3_OD) δ: 7.55 (2H, d, *J* = 15.6 Hz, H-7′, 7″), 7.03 (1H, d, *J* = 2.4 Hz, H-2′), 7.02 (1H, d, *J* = 1.8 Hz, H-2″), 6.91 (1H, dd, *J* = 1.8 Hz, 8.4 Hz, H-6′), 6.88 (1H, dd, *J* = 1.8 Hz, 8.4 Hz, H-6″), 6.76 (1H, d, *J* = 7.8 Hz, H-5′), 6.73 (1H, d, *J* = 8.4 Hz, H-5″), 6.28 (1H, d, *J* = 15.6 Hz, H-8′), 6.25 (1H, d, *J* = 15.6 Hz, H-8″), 5.63 (1H, br s, H-3), 5.12 (1H, br s, H-4), 4.38 (1H, br s, H-5), 2.16 (2H, m, H-6), 2.09 (2H, m, H-2). ^13^C-NMR (125 MHz, CD_3_OD) δ: 176.9 (C-7), 168.6 (C-9′, 9″), 149.7 (C-4′, 4″), 147.4 (C-7′, 7″), 146.9 (C-3′, 3″), 127.8 (C-1′), 127.7 (C-1″), 123.3 (C-6′), 123.2 (C-6″), 116.5 (C-5′, 5″), 115.3 (C-2′), 115.1 (C-2″), 115.0 (C-8′, 8″), 75.9 (C-1), 75.3 (C-4), 70.2 (C-3), 67.0 (C-5), 39.7 (C-2), 37.8 (C-6). The structure was assigned by ^1^H-^1^H COSY, HMQC and HMBC. Comparing the data with reference [29], the obtained compound was identified as 3,4-*O*-dicaffeoylquinic acid.

*Hyperoside* (**7**, Figure 5E): ESI-MS, *m/z* 465.0 [M + H]^+^, 463.0 [M − H]^−^. ^1^H-NMR (600 MHz, CD_3_OD) δ: 7.69 (1H, d, *J* = 1.8 Hz, H-2′), 7.58 (1H, dd, *J* = 1.8, 7.8 Hz, H-6′), 6.85 (1H, d, *J* = 8.4 Hz, H-5′), 6.39 (1H, d, *J* = 2.4 Hz, H-8), 6.19 (1H, d, *J* = 1.8 Hz, H-6), 5.25 (1H, d, *J* = 7.8 Hz, H-1″). ^13^C-NMR (125 MHz, CD_3_OD) δ: 179.6 (C-4), 166.1 (C-7), 163.1 (C-5), 158.5 (C-2, 9), 149.9 (C-4′), 146.0 (C-3′), 135.6 (C-3), 123.1 (C-6′), 119.8 (C-1′), 117.6 (C-5′), 116.0 (C-2′), 105.7 (C-10), 104.2 (gal C-1), 99.9 (C-6), 94.7 (C-8), 78.5 (gal C-5), 75.8 (gal C-3), 71.2 (gal C-2), 69.9 (gal C-4), 62.6 (gal C-6). Comparing the data with reference [30], the obtained compound was identified as hyperoside. 

*3,5-O-Dicaffeoylquinic acid* (**8**, Figure 5F): ESI-MS, *m/z* 539.3 [M + Na]^+^, 515.3 [M − H]^−^. ^1^H-NMR (600 MHz, CD_3_OD) δ: 7.61 (1H, d, *J* = 15.6, H-7′), 7.57 (1H, d, *J* = 16.2, H-7″), 7.07 (1H, d, *J* = 2.4, H-2′), 7.06 (1H, d, *J* = 2.4, H-2″), 6.97 (2H, m, H-6′, 6″), 6.78 (2H, d, *J* = 7.8, H-5′, 5″), 6.36 (1H, d, *J* = 15.6, H-8′), 6.27 (1H, d, *J* = 15.6, H-8″), 5.43 (1H, m, H-5), 5.40 (1H, m, H-3), 3.97 (1H, d, *J* = 4.2, H-4), 2.31 (1H, br s, H-6α), 2.22 (2H, m, H-2), 2.16 (1H, m, H-6β). ^13^C-NMR (125 MHz, CD_3_OD) δ: 176.3 (C-7), 166.9 (C-9′), 167.5 (C-9″), 148.2 (C-4′), 148.1 (C-4″), 145.8 (C-7′), 145.6 (C-7″), 145.4 (C-3′, 3″), 126.5 (C-1′), 126.4 (C-1″), 121.6 (C-6′, 6″), 115.0 (C-5′, 5″), 114.2 (C-8′), 113.7 (C-8″), 113.8 (C-2′), 113.7 (C-2″), 73.5 (C-1), 71.2 (C-5), 70.7 (C-3), 69.3 (C-4), 36.4 (C-2), 36.0 (C-6). The structure was assigned by ^1^H-^1^H COSY, HMQC and HMBC. Comparing the data with reference [31], the obtained compound was identified as 3,5-*O*-dicaffeoylquinic acid.

*4,5-O-Dicaffeoylquinic acid* (**9**, Figure 5G): ESI-MS, *m/z* 517.3 [M + H]^+^, 515.3 [M − H]^−^. ^1^H-NMR (600 MHz, CD_3_OD) δ: 7.59 (1H, d, *J* = 16.2, H-7′), 7.51 (1H, d, *J* = 15.6, H-7″), 7.02 (1H, d, *J* = 1.8, H-2′), 7.00 (1H, d, *J* = 1.8, H-2″), 6.91 (1H, d, *J* = 7.8, H-6′), 6.90 (1H, d, *J* = 8.4, H-6″), 6.74 (2H, m, H-5′, 5″), 6.29 (1H, d, *J* = 15.6, H-8′), 6.19 (1H, d, *J* = 15.6, H-8″), 5.61 (1H, br s, H-5), 5.12 (1H, d, *J* = 7.2, H-4), 4.38 (1H, br s, H-3), 2.28 (3H, m, H-2, 6α), 2.14 (1H, m, H-6β). ^13^C-NMR (125 MHz, CD_3_OD) δ: 175.3 (C-7), 167.1 (C-9′), 166.8 (C-9″), 148.2 (C-4′, 4″), 146.3 (C-7′), 146.2 (C-7″), 145.3 (C-3′, 3″), 126.2 (C-1′, 1″), 121.7 (C-6′, 6″), 115.0 (C-5′, 5″), 113.7 (C-2′, 2″), 113.3 (C-8′), 113.2 (C-8″), 74.8 (C-1), 74.6 (C-4), 68.4 (C-3), 67.9 (C-5), 38.4 (C-6), 37.2 (C-2). The structure was assigned by ^1^H-^1^H COSY, HMQC and HMBC. Comparing the data with reference [31], the obtained compound was identified as 4,5-*O*-dicaffeoylquinic acid.

## 4. Conclusions

**Figure 6:**
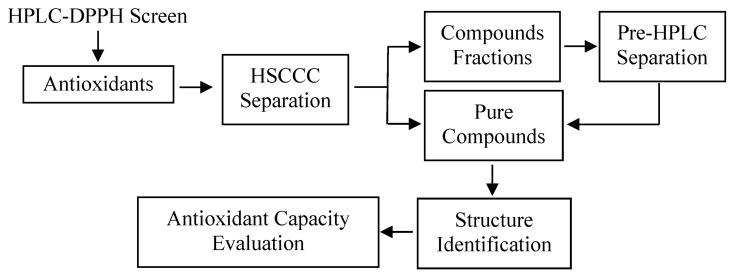
The diagram of the procedure.

Our study demonstrated an efficient method using HSCCC and prep-HPLC guided by DPPH-HPLC experiments for screening and preparative separation of flavonoid glycosides and caffeoylquinic acid derivatives from leaves of *L. japonica* (Figure 6). As shown in Figure 2, it was difficult to separate peaks **1**–**9** using preparative HPLC directly due to their similar polarity. Using HSCCC followed by prep-HPLC, nine high purity flavonoid glycosides and caffeoylquinic acid derivatives could be obtained from the *n*-butanol extract of leaves from *L. Japonica*.

It was an efficient method to achieve successful separation compounds with similar polarity, and a superior separation method for flavonoid glycosides and caffeoylquinic acid derivatives over other chromatographic methods for complementary action between these two methods. This method might be an excellent technique for rapid preparative isolation of other complicated natural products.

## Figures and Tables

**Table 1:** Antioxidant activities of crude extracts and nine isolated compounds from leaves of *L. japonica* in DPPH assay.

Samples	DPPH (IC_50_, μg/mL) ^a^
*n*-Butanol extract of *L. japonica*	11.2
Chlorogenic acid (**1**)	9.1
Lonicerin (**2**)	6.3
Rutin (**3**)	10.7
Rhoifolin (**4**)	6.7
Luteoloside (**5**)	5.9
3,4-*O*-Dicaffeoylquinic acid (**6**)	11.4
Hyperoside (**7**)	7.2
3,5-*O*-Dicaffeoylquinic acid (**8**)	9.3
4,5-*O*-Dicaffeoylquinic acid (**9**)	11.8
Ascorbic acid ^b^	6.4
Vitamin E ^b^	9.5

**^a^** Each value is presented as mean ± SD (*n* = 3); ^b^ Used as control.

**Table 2:** The *K_D_*-values of target components measured in different solvent systems.

Solvent System	*K*_D_-Values of Compounds 1–9								
	1	2	3	4	5	6	7	8	9
System A	0.53	0.52	0.47	1.03	1.66	1.83	0.85	4.48	3.08
System B	0.98	1.01	0.92	1.93	3.40	2.01	1.26	8.55	7.30
System C	1.67	1.85	1.56	5.34	8.16	4.28	2.46	13.26	11.02
System D	1.10	0.99	1.15	2.13	3.43	4.40	4.16	5.32	5.17
System E	2.59	2.77	2.52	3.81	5.27	6.96	6.71	8.20	7.64
System F	1.89	1.94	1.85	2.88	4.14	5.11	4.78	6.24	5.78

Systems A, B and C were *n*-hexane/*n*-butanol/methanol/water (1:3:1:4, *v*/*v*), (1:4:1:4, *v*/*v*) and (1:4:1:5, *v/v*), respectively. Systems D, E and F were methyl *tert*-butyl ether/*n*-butanol/acetonitrile/water (0.5% acetic acid) (2.5:1.5:1:5, *v/v*), (1.5:2.5:1:5, *v/v*) and (2:2:1:5, *v/v*), respectively.

**Table 3:** Processing conditions for the preparative HPLC separations of sub-fractions F1, F4 and F5. (A: MeOH, B: 0.3% acetic acid in water, *v/v*).

Sample	Initial (%)	End (%)	Run Time (min)	Flow Rate (mL/min)
F1	A/B (45:55)	A/B (45:55)	25	10
F4	A/B (30:70)	A/B (60:40)	30	10
F5	A/B (38:62)	A/B (48:52)	20	10
